# The Impact of the COVID-19 Pandemic on Orthopaedic Trauma Surgery in a District General Hospital in the United Kingdom

**DOI:** 10.7759/cureus.53928

**Published:** 2024-02-09

**Authors:** Govind S Chauhan, Jasprit Kaur, Awais Habeebullah, Varun Dewan, Gopalakrishna Pemmaraju

**Affiliations:** 1 Orthopaedics, The Royal Wolverhampton NHS Trust, Wolverhampton, GBR

**Keywords:** mortality, fracture, orthopaedics, trauma, pandemic, covid-19, coronavirus

## Abstract

Introduction

The 2020 COVID-19 pandemic led to a national lockdown and a major reorganization of healthcare services in the United Kingdom. The center where the study was done was one of the worst affected hospitals in the United Kingdom at the outset of the pandemic. Our study evaluates the impact of the pandemic and national lockdown on the outcomes for patients undergoing orthopedic trauma surgery.

Methods

We prospectively identified all patients undergoing orthopedic trauma surgery in the unit from 1st March 2020 to 31st May 2020. We recorded demographics, diagnoses, COVID-19 infection status, length of stay, and mortality. This was compared with a comparative group in the same period in 2018 and 2019.

Results

There was a significant reduction in the number of orthopedic trauma surgery cases (318) performed in 2020 compared to 2019 (423 cases, p<0.001) and 2018 (444 cases, p<0.001). The mean time from injury to presentation was 3.6 days, with 40 patients (12.6%) presenting more than one week after injury.

The 30-day mortality was 8.2%, and the six-month mortality was 15.1%, with both significantly higher than in 2018 (p<0.001) and 2019 (p<0.001). COVID-19 testing was positive in 39% of patients, with 30-day mortality in this group at 37%, rising to a 53% six-month mortality. No patients under the age of 50 years old died.

The majority of admissions (51%) were due to falls at home. The second most common mechanism was Do-It-Yourself (DIY) injuries. Road traffic accidents accounted for 2%.

Conclusion

There were significantly fewer cases of orthopedic trauma surgery during the first wave of the COVID-19 pandemic compared to the same period in previous years. The type of trauma also showed low numbers of high-energy and sporting injuries as a result of the national lockdown. Patients undergoing orthopedic trauma surgery who tested positive for COVID-19 had significantly higher 30-day mortality than those without COVID-19, and this increased mortality persisted to six months post-operatively. However, patients under 50 years old appear to be at low risk of death.

## Introduction

COVID-19, an illness caused by the Severe Acute Respiratory Syndrome Corona Virus 2 (SARS-CoV-2), was first identified in China in December 2019 before becoming a global pandemic in early 2020. In the United Kingdom, the first cases were identified at the end of January [1], with the first death on the 5th March 2020 [2]. A national lockdown was enforced on 23rd March 2020 [3] to reduce the spread of the virus and reduce the burden on the National Health Service.

COVID-19 results in varying severity of illness, and several risk factors for severe illness have been identified. These include older age and a history of cardiovascular disease, chronic renal disease, diabetes mellitus, and chronic respiratory disease [4,5]. Most patients will have a milder form of the illness [4].

A large proportion of patients requiring fracture surgery are older than 65, and many have pre-existing co-morbidities [6]. Elderly trauma patients are already known to have worse outcomes than the young [7,8]. Younger patients tend to present after higher energy injuries or injuries in the workplace, and the national lockdown may lead to a reduction in these types of injuries [9]. Early data from China showed that patients with COVID-19 who sustained a fracture had a more severe illness than those without a fracture [10].

Our center is a district general hospital in the United Kingdom and is also a trauma unit. It was one of the worst affected hospitals in the United Kingdom at the outset of the pandemic [11]. Our study aims to evaluate the impact of the pandemic and the national lockdown on the outcomes for patients undergoing orthopedic trauma surgery in our unit.

## Materials and methods

We identified all patients undergoing orthopedic trauma surgery in our unit from 1st March 2020 to 31st May 2020. This included both bony and soft tissue procedures.

Patient demographics, the mechanism, and the date of injury were recorded. The date of the decision for surgery was also noted. The intraoperative details were taken from the anesthetic charts and surgical operation notes. COVID status was noted. In our unit, COVID-19 infection was confirmed by quantitative RT-PCR analysis. Patients were followed through to discharge, and mortality data was reviewed at six months.

We also retrospectively reviewed the trauma admissions over the same period in 2019 and 2018 as comparative groups, focussing on length of stay and mortality. The study design is akin to a case-control study. Categorical data was analyzed with a chi-squared test. Continuous data was analyzed with the Mann-Whitney U Test.

## Results

In the two-month study period, there were 318 emergency orthopedic trauma surgery cases. This compared to 423 cases in 2019 and 444 in 2018, showing a significant reduction (p<0.001) in the number of emergency orthopedic trauma cases.

The median age of admission was 66.3 years (range 5 to 100 years), with patients older than those in 2019 (63.0 years, p=0.0117) and 2018 (62.3 years, p=0.0349).

The mean time from injury or onset of symptoms to presentation was 3.6 days (range 0 - 46 days). Of the 318 patients, 216 (67.9%) presented on the day or the day after the onset of symptoms. Forty patients (12.6%) presented more than one week after the onset of their symptoms, and nine patients (2.8%) presented more than one month after their injury.

The median time to surgery from the operation decision was 1 day (range 0 - 26 days). The length of stay showed a positively skewed distribution for all years, with median length of stay in 2020 being 5 days (interquartile range (IQR) 1 to 18 days) compared to 2 days (IQR 0 - 14) in 2019 and 2 days (IQR 0 - 12) in 2018.

As shown in Table 1, comparing all patients, the results showed a significantly higher mortality at both 30 days (p <0.001) and six months (p <0.001) when compared to 2019 and 2018. The 30-day mortality in 2020 was 8.2%, compared to 3.3% in 2019, and 3.2% in 2018. The six-month mortality was 15.1% in 2020, compared to 7.6% in 2019, and 9.0% in 2018.

**Table 1:** Age, length of stay, and mortality of patients undergoing non-elective orthopedic surgery from 1st March to 31st May in 2020, 2019, and 2018

	2018	2019	2020
Number of patients	444	423	318
Median Age (Years)	62.3	63.0	66.3
Age Range (Years)	2 – 101	1 – 99	5 – 100
Median Length of stay (days)	2	2	5
Length of stay – Interquartile Range (days)	0 – 12	0 – 14	1 – 18
30-day mortality	3.2%	3.3%	8.2%
Six-month mortality	9.0%	7.6%	15.1%

COVID-19

COVID-19 testing was carried out on 209 patients, with 43 (39%) testing positive. Table 2 shows the mortality in these patients compared with the previous years. Out of the positive patients, 16 (37%) died within 30 days of their positive COVID result, rising to 23 (53%) deaths by six months. This was significantly higher at both 30 days and six months when compared to those who tested negative (p<0.001), those who were not tested (i.e. asymptomatic patients) (p<0.001), those having surgery in 2019 (p<0.001), and those having surgery in 2018 (p<0.001). The mean age of those who died was 81.0 years, and of those who were still alive at six months was 82.3 years (p<0.001). None of the 112 patients under the age of 50 years died in the 2020 study period, regardless of their COVID-19 status.

**Table 2:** Mortality of patients undergoing non-elective orthopedic surgery in 2018, 2019, and 2020 and the effect of COVID-19 status on mortality.

	Number of patients	30-day mortality	6-month mortality
2020 COVID-19 positive	43	16 (37%)	23 (53%)
2020 COVID-19 negative	166	11 (7%)	22 (13%)
2020 Not tested (asymptomatic)	109	3 (3%)	3 (3%)
2020 Total	318	30 (9%)	48 (15%)
2019	423	14 (3%)	32 (8%)
2018	444	14 (3%)	40 (9%)

Mechanism

Table 3 shows the mechanism of injury for the patients in the 2020 cohort. The majority (51%) of admissions were due to falls at home. Do-It-Yourself (DIY) injuries at home accounted for 10% of cases, and infection for 9%. Cycling injuries (7%), sports injuries (5%) and road traffic-related injuries (2%) represented a small proportion of admissions.

**Table 3:** Mechanism of injury for patients undergoing non-elective orthopedic surgery during the first wave of the coronavirus pandemic.

Mechanism of injury	Number of patients (%)
Fall at home	163 (51%)
DIY & lacerations at home	31 (10%)
Infections	30 (9%)
Cycling injury	22 (7%)
Fall outside of home	20 (6%)
Sports	17 (5%)
Road traffic accident	7 (2%)
Assault	6 (2%)
Injury at work	5 (2%)
Animal bite	4 (1%)
Chronic instability	4 (1%)
Post-op complication	3 (1%)
Spinal emergency	3 (1%)
Cancer	1 (0.3%)
Inpatient fall	1 (0.3%)
School accident	1 (0.3%)

Diagnosis

The most common type of injury was a proximal femoral fracture (37%). There was a high proportion of wounds and lacerations (14%). There were relatively few other lower limb long bone fractures (4% femur, 2% tibias). The full list of diagnoses is shown in Table 4.

**Table 4:** Diagnosis of patients undergoing non-elective orthopedic surgery during the first wave of the 2020 coronavirus pandemic.

Diagnosis	Number of patients
Proximal femoral fracture	116 (37%)
Wounds / Laceration	45 (14%)
Infection	30 (9%)
Ankle fracture	21 (7%)
Upper limb fracture (excluding hand and distal radius)	15 (5%)
Distal Radius fracture	15 (5%)
Hand fractures	14 (4%)
Femoral fracture	12 (4%)
Dislocation of total hip replacement	12 (4%)
Paediatric fracture	12 (4%)
Tibial fracture (excluding ankle)	6 (2%)
Shoulder dislocation	6 (2%)
Soft tissue knee injury	4 (1%)
Foot fracture	3 (1%)
Spinal emergency (not fracture)	3 (1%)
Failure of metalwork	2 (1%)
Peri-prosthetic fracture	2 (1%)

## Discussion

The most important finding of our study is that our data shows a reduction in overall trauma cases in our region during the 2020 first wave of the COVID-19 pandemic in comparison to previous years. This was expected due to the national lockdown and people staying home. Similar findings have been reported in Scotland, Spain, Ireland and Australia [9,12-14]. The distribution of injuries showed ongoing low-energy trauma from injuries at home (proximal femoral fractures, distal radius fractures, ankle fractures) with a low incidence of high-energy injuries. There were very few sports injuries (5%) and road traffic accident-related injuries (2%), which is a potential positive impact of the lockdown. This suggests that not only did the lockdown reduce the social interaction of patients, which was its main purpose of reducing the spread of the highly contagious viral respiratory disease, but it may also have reduced the number of high-energy trauma admissions and thus freed up capacity within the hospital to accommodate the high number of patients being admitted with COVID-19. There were similar findings by Campbell, who found an 18% reduction in open fracture admissions in London, England, during the first wave of the COVID-19 pandemic [15]. Murphy also showed a 26% reduction in surgical procedures during the pandemic in Gloucester, England [16].

The majority of patients sustained their injuries in their own homes. The commonest presentation was falls in the patient’s own home, followed by DIY injuries in their own home. This is consistent with the trend widely reported in the media of increased sales in the DIY sector [17]. The national lockdown led to most people staying at home during this time, with only essential reasons to leave the home permitted. For future pandemic planning, a consideration to advise the public to avoid household activities that risk significant injury may be beneficial in reducing the burden of household injuries on healthcare systems.

We had anticipated that our length of stay would have been shorter during the COVID-19 pandemic due to a drive to treat patients with day-case surgeries. However, we found an increase in the length of stay compared to previous years. There was a large positive skew to the data, with a high number of day-case and one-night stays but with a long tail. Stays over 14 days were seen in 94 patients (30.0%), with the majority of these being elderly. One potential explanation for this is the difficulties in discharging patients due to the COVID-19 protocols in place which required isolation periods before discharge to care facilities. Our findings differ from those reported by Wignall, who showed a shorter length of stay amongst their hip fracture cohort [18].

Numerous patients had a delayed presentation to the hospital following their injury, with 102 patients (32.1%) presenting later than day two after their injury, 40 patients (12.6%) more than one week, and 9 (2.8%) patients presenting more than one month after their injury. Notably, some of these patients cited avoiding the hospital due to a fear of exposure to COVID-19, while some cited avoiding the hospital as they felt hospitals were overwhelmed and did not want to burden them further.

A significant finding from our study is the high mortality rate among patients testing positive for COVID-19 around the time of their injury and surgery. Patients undergoing orthopedic emergency or trauma surgery who tested positive for COVID-19 had a 30-day mortality of 37% and a six-month mortality of 53%. Those who tested negative for COVID-19 in the same period had a 30-day and six-month mortality of 7% and 13% respectively. These mortality rates in COVID-negative patients are higher than the mortality rates before the pandemic. Although our study is unable to give causation, potential factors include the impact of the reorganization of services and staff, a reduction in theatre capacity, reduced availability of beds in higher-level care, or a longer length of stay. The COVID-Surg Collaborative showed a 23.8% 30-day mortality in their pan-specialty study, and further detail in their results showed that patients undergoing orthopedic surgery had a 71.2% 30-day mortality [19]. Mi also reported a higher mortality rate among COVID-19 patients with fractures than those without fractures [10].

We acknowledge that our data is from the first wave of the COVID-19 pandemic and predates the use of vaccinations and newer treatment techniques; thus, current mortality rates for patients undergoing orthopedic surgery with COVID-19 are anticipated to be lower.

## Conclusions

In conclusion, our study has shown that there were significantly fewer cases of orthopedic trauma surgery during the first wave of the COVID-19 pandemic compared to the same period in previous years. The type of trauma also showed low numbers of high-energy and sporting injuries, which could be a result of the national lockdown. Patients undergoing orthopedic trauma surgery who tested positive for COVID-19 had significantly higher 30-day mortality than those without COVID-19, and this increased mortality persisted to six months post-operatively. However, patients under 50 years old appear to be at low risk of death.
